# Where are they working? A case study of twenty Cuban-trained South African doctors

**DOI:** 10.4102/phcfm.v11i1.1977

**Published:** 2019-08-21

**Authors:** Munirah Motala, Jacqueline van Wyk

**Affiliations:** 1 School of Medicine, University of KwaZulu-Natal, Durban, South Africa

**Keywords:** doctor shortages, primary health care, Cuban-trained, South African, medical programme

## Abstract

**Background:**

The year 2017 marked the 21st anniversary of the South African Cuban Medical Collaboration (SACMC) programme that offers disadvantaged South African (SA) students an opportunity for medical training in Cuba. Graduates are expected to return to practice at a primary care level in rural communities; however, little is known about the professional trajectories and career choices of graduates from the programme.

**Aim:**

This study explored the reasons why students enrolled in the programme, their professional and career choices as graduates and their career intentions.

**Setting:**

The study setting was the whole of SA although participants were primarily drawn from KwaZulu-Natal.

**Methods:**

An exploratory, qualitative case study used a purposive sampling strategy to gather data through semi-structured interviews from participants.

**Results:**

Graduates (*N* = 20) of the SACMC programme were all practicing in local SA settings. Participants preferred the SACMC programme as it offered them a full scholarship for medical training. Nineteen doctors had fulfilled their obligation to work in rural areas. Thirteen doctors are engaged in primary healthcare practice, either as private practice generalists or as public service medical officers. Three doctors had completed specialty training: one doctor was training towards specialisation, one doctor was employed at national government and two doctors were employed as medical managers. At the time of the study, 11 doctors were practicing in rural locations and 19 had indicated a long-term intention to work and live within South Africa.

**Conclusion:**

The participants of this study who graduated from the SACMC programme are fulfilling their obligations in rural communities. They all intend to contribute to the SA medical workforce in the long-term.

## Introduction

South Africa’s eight medical schools produce approximately 1600 doctors per year,^1^ a number that is insufficient for the medical needs of South Africa’s population of more than 50 million people.^2^ The South African Department of Health (DoH) has implemented various interventions to increase and retain the services of doctors in rural areas. Some interventions include rural allowances, improved working conditions and the introduction of compulsory community service.^3^ The employment of foreign-trained medical doctors to rural practice also provided an interim solution to human resource challenges. Another strategy to address the shortage of doctors in rural areas rests on the country’s ability to produce more doctors.^4^

Cuba, on the other hand, has successfully trained enough medical doctors to accommodate the needs of its own population and to supply healthcare personnel to alleviate the shortage of doctors in other countries. The 25 Cuban medical schools produce an annual average of about 11 000 doctors.^5^ Cuba’s international collaboration projects provided disaster relief after the earthquake in Haiti,^6^ supplied Cuban-trained doctors to Algeria,^7^ improved access to health for the poor and visually impaired in Venezuela^8^ and have alleviated the shortage of doctors to the most deprived communities in rural Brazil.^9^ Cuba’s collaboration includes medical training to students from around the world. The Latin American School of Medicine (ELAM) established in 1999^10^ provides medical training to students from disadvantaged communities who have been identified by a social organisation, such as a church that is active in the community from the students’ country of origin.

South Africa has been a major beneficiary of the Cuban medical internationalism programme. The initiative, launched between 1996 and 2002, started with the placement of more than 450 Cuban-origin doctors and medical lecturers who were deployed for service to South Africa^11^ and it extended to the enrolment of South African (SA) students for medical training under the Nelson Mandela-Fidel Castro collaboration. The graduates from the South African Cuban Medical Collaboration (SACMC) receive medical training in Cuban settings on condition that they return and practice in South Africa’s public sector for a period that equates to the time that they had benefitted from the scholarship. The collaboration agreement aims to provide healthcare to communities where local indigenous populations had previously lacked access to healthcare and is similar to other Cuban-based international programmes to train doctors such as the Brazil and South Pacific island collaborations.^9,12^ Students on the SACMC programme are selected specifically from rural areas and preference is given to those who had limited access to tertiary studies because of socio-economic constraints. Research has indicated that a rural background was a determining factor influencing the attitudes, intentions and preferences of medical graduates to practice in rural settings.^13,14^ The successful implementation of the primary healthcare (PHC) model in Cuba has furthermore led to the eradication of infectious diseases, an achievement that locates Cuba in terms of its health indicators, as comparable to many high-income countries.^15^

The SACMC programme was conceptualised for two purposes, that is, to train more rural-origin SA students in Cuba to supplement the limited training capacity that is produced by SA institutions and for graduates to use their medical knowledge of PHC to transform the health of the local SA communities.^16^ The SACMC programme is therefore aligned to the newly formulated National Health Insurance bill (NHI), which requires the education of more doctors, to address the historical and inequitable healthcare situation in South Africa.^17^ The NHI scheme is meant to facilitate universal access to healthcare services to all citizens irrespective of their socio-economic status. Despite some confusion over the goals of the NHI,^18^ its successful implementation depends on the availability of qualified practitioners to work in the rural, PHC and other sectors of the country.^2^

The SACMC is also aligned to South Africa’s plans towards the re-engineering of the PHC system. A key policy reform that underpins the re-engineering of the PHC system seeks to shift the philosophy and practice of the current PHC system, which is largely based on a curative and individualistic model to incorporate a more community-focused, proactive and preventative model.^19^ In this way, it is believed that graduates from the SACMC, who receive their medical training in the contexts of a well-functioning PHC system in Cuba, would actively contribute to the transformation of the SA healthcare workforce.

Many of the detractors of the SACMC programme consider the intervention a waste of money that could have increased the training capacity of local medical schools.^5,20^ The Cuban medical training programme has also been criticised for being misaligned to local medical programmes and is therefore adding to the students’ difficulty to graduate from local medical programmes^21^ and to adjust, integrate and succeed in the SA health care system.^5,22^ The SACMC programme has expanded exponentially in the last 7 years with about 1000 undergraduate medical students expected to complete their training agreements in the next 5 years.^5^ Given the significant lack of academic published literature on the career choices of graduates of the SACMC programme, this study explored the reasons why rural-origin SA students chose to study medicine in Cuba, their professional and career choices after graduation and their career intentions for the foreseeable future.

## Methods

### Study design

An exploratory, descriptive case study constituting a purposive sample of 20 SA doctors who had enrolled on the SACMC programme between 1997 and 2007 was conducted.^23^ The study involved the collection of qualitative data through semi-structured interviews.

### Setting

The SACMC programme is a collaboration between the SA and Cuban governments which dates back to 1996 when the first students were enrolled. The graduates from the SACMC receive medical training in Cuban settings on condition that they return and practice in South Africa’s public sector for a period that equates to the time that they had benefitted from the scholarship. The collaboration agreement aims to provide healthcare to communities where local indigenous populations had previously lacked access to healthcare. The researchers are both affiliated with a university that is involved in the training of Cuban-trained students upon their re-entry to SA and have conducted educational research to support students upon joining the final year at the institution.

### Sampling

A provincial representative from the DoH was approached for permission to access a database of students who entered the SACMC programme. An e-copy of the database (*N* = 104) was received (November 2016) with the personal particulars of the students and the mobile telephone number of those who had enrolled from KwaZulu-Natal (KZN). The resource also included the details of the hospitals where the graduates had been placed for internship. Efforts to contact the graduates from the list showed that most of the mobile numbers were no longer in use but some of the graduates could be traced through the healthcare facility. Those who were contacted were informed about the study and permission was sought for their participation in the study.

Informed by the aim of the study to explore the career decisions of graduates from the SACMC, the researchers were interested in participants who had completed the training and compulsory internship and community service period. A purposive sample of graduates who had undergone training during the first decade of the SACMC programme and who had returned to South Africa from 2002 to 2012 were identified as eligible participants. All of the participants in the current case study (*n* = 20) had enrolled on the SACMC programme from 1997 to 2007. Four of the eligible participants (*N* = 104) who were originally listed on the database were deceased at the time of data collection, a substantial number (*n* = 61) did not answer when called on the listed mobile number, some refused (*n* = 16) to participate and 14 participants from the database eventually agreed to be interviewed. Using a snowballing strategy, the researchers requested those who had agreed to the interview to identify other suitable participants who had graduated from the programme during the specified period.^24^ This strategy yielded the additional participants.

The majority (*n* = 19) of the participants were interviewed telephonically to accommodate their work schedules and one was interviewed in a face-to-face session. The purposive sampling strategy allowed for the selection of the participants who had trained on the SACMC and who had sufficient time since their return to pursue a career in South Africa.^23^

### Data collection

Data were collected with the help of a researcher-administered interview guide. The interviews took an average of 40 minutes to complete. Data were collected between 13 May 2017 and 09 August 2017. For the purpose of this study, participants were asked about the general geographic location, that is, whether they were practicing in an urban, semi-urban or rural area. A rural area was defined as a location of more than 2 hours travel (approximately 240 km) from an urban area, while a semi-urban area was defined as a destination that was less than 2 hours of travel by car from an urban area.^25^

Data collection through interviews requires the building of trust between the researcher and the interviewees.^26^ This was achieved through a method whereby the researcher firstly established contact with the participants, the study and approach were explained, and an interaction with each participant followed. Participants were called and informed of the study and their right to withdraw at any stage. The researcher then obtained an email address and provided each participant with information about the nature and aim of the study. The shared information included an overview of the issues to be discussed, informed consent and anonymity were explained, and they were assured of the researchers’ adherence to ethical principles in the conduct of the study. Permission was obtained to audio-record the interviews which added to the accuracy and richness of the data. The interviews were transcribed in full. The data collection process was stopped when saturation was reached. This was after 20 of the eligible participants (*N* = 104) had been interviewed. There is no one-size-fits-all method to reach data saturation which means that the interviews yielded no new data, no new themes, no new coding and yielded sufficient information to replicate the study.^27^

### Data analysis

The qualitative data were analysed deductively^28^ in response to each of the research objectives of the study. The four steps in the analysis process included firstly organising the data by going back to the interview guide, identifying the questions and then organising the data in response to each research question.^29^ The second step involved each of the authors independently identifying from the transcripts, the concepts relating to each research question, thirdly the concepts were coded into categories and finally the categories identified in step 3 were grouped into overarching themes that answered the research objectives. The authors had subsequent meetings to discuss and agree on the process of data analysis and the reporting of the final themes, that is, they followed a process of external validation of the categories and themes.^29^ Independent analysis by two researchers was used to ensure the credibility of data analysis.

### Ethical considerations

Ethical approval was obtained from the Humanities and Social Science Research Ethics Committee (HSS/1066/016D) of the University of KwaZulu-Natal (UKZN) and gatekeeper permission was obtained from the KZN-DoH. Individual consent was obtained from all participants after they had been informed about the nature and purpose of the study; their rights in terms of voluntary participation; and after assuring them of confidentiality and anonymity during the research process.

## Findings

### Description of the participants

A total number of 6 women and 14 men agreed to the interview. The average age of the participants was 39 years. All participants were born between 1971 and 1987 with their ages ranging between 30 and 46 years. At the time of data collection, 13 doctors were married, six were single and one was divorced. All the doctors originated from rural areas of four provinces, namely KZN, Mpumulanga (MP), the North West province (NW) and Limpopo (LP). The participants all went to Cuba between 1997 and 2007, the period coincides with the first 10 years of the SACMC programme. All participants had returned to South Africa between 2002 and 2012 and had graduated from three Cuban-based medical schools. They had completed their training at seven of the eight local SA medical schools, as shown in Table 1.

**TABLE 1:** Biographical details of participants.

Variables	No. of participants
A: Home province	
KZN	14
MP	3
NW	2
LP	1
B: Home language	
isiZulu	13
Siswati	3
Tshwana	2
Isitonga	1
English	1
C: Cuban-based institution	
Cienfugos	9
Santa Clara	8
Santa Spiritus	3
D: South Africa-based institution	
University of KwaZulu-Natal (UKZN)	10
Medical University of South Africa (Medunsa)	2
University of Stellenbosch (US)	2
University of the Witwatersrand (Wits)	2
University of Pretoria (UP)	2
University of Cape Town (UCT)	1
Walter Sisulu University (WSU)	1

KZN, KwaZulu-Natal; MP, Mpumulanga; NW, North West; LP, Limpopo; SA, South African.

### Reasons for enrolling on the programme

All the doctors were asked about their reasons for choosing the SACMC programme. They revealed that the programme had offered them full financial support to complete the medical qualification. The participants explained that funding for tertiary studies was relatively inaccessible to them as students from poor socio-economic households and under-resourced secondary schooling backgrounds. As indicated in the quotes below, the graduates thus viewed the SACMC as an opportunity to further themselves.

‘Well quite honestly I didn’t choose to study in Cuba, it was a question of I didn’t have money and I wanted to study medicine and the only scholarship I did secure was offered to go to Cuba.’ (Doctor E, male, 36, 14 June 2017)

‘Yeah it was purely, for me personally it was financial reasons.’ (Doctor F, male, 38, 21 June 2017)

‘I think it was more of a career opportunity that was given to me coming from the rural area and um not being advantaged enough to get to into institutions around South Africa, then I just grabbed that opportunity.’ (Doctor S, male, 37, 11 July 2017)

Most of the early graduates from the programme were furthermore from a pool of students who had been unsuccessful as applicants to local universities. Given their rural and disadvantaged upbringing and improbable access to funding, the full scholarship to study medicine abroad had offered them an option to make something of their lives.

‘As a young child, growing in the rural area you always have aspirations, you find that, background circumstances don’t allow you to think that big. It was always my dream to go to university, I didn’t think I would ever get a chance because of my family background. So immediately after finishing my matric, based on my results that were good, I saw this opportunity, I just applied so that’s how I ended up in Cuba, but I would have gone to any university in South Africa if I had the chance as well.’ (Doctor H, male, 35, 22 June 2017)

### Reasons for choosing medicine as a career

The thematic analysis revealed four themes in response to the question that explored why participants had chosen medicine as a career. The themes included medicine as a serendipitous opportunity, having had a childhood dream, altruism or a desire to help people and the community and exposure to a role model. These themes are illustrated and supported by quotes below.

#### Opportunity

‘I would say that it was not really a matter of choice, I had, after I had completed matric I started applying for different courses and stuff. Unfortunately I didn’t get accepted with anything, and then the Cuban program came up …’ (Doctor C, male, 34, 05 June 2017)

‘One morning I switched on the radio Ligwalagwala l FM basically a radio station from Mpumalanga that were giving bursaries for medicine in Cuba and then I decided after some contemplation why not, and fortunately I was successful, then January of the following year I went to Cuba …’ (Doctor O, female, 41, 05 July 2017)

For some graduates, the opportunity to study medicine was viewed as ‘fate’ or a rare opportunity as a lack of counselling and insufficient career guidance at secondary school level did not really help in the career decision-making process.

‘Yeah as I am saying, um we did not receive the proper guidance as to what exactly you would like to choose so what you would like to become when you would finish. We were given those studies and then on the studies in which we were given, that you passed you would be given careers that you fit in in. So we were not so much exposed to medicine in our schooling …’ (Doctor C, male, 34, 05 June 2017)

‘I didn’t, I say it was fate, fate chose it for me. I was a businessman by then and I was running a spaza shop and my mother kept on telling it wasn’t the way to go for me …’ (Doctor D, male, 40, 13 June 2017)

‘Well, it was the only opportunity that I had to study medicine, ya, it was the only opportunity that I had, so because I always wanted to study medicine but I didn’t have the money to go to the other universities.’ (Doctor T, female, 42, 09 August 2017)

#### Childhood dream of becoming a doctor:

As indicated, a few of the doctors expressed having had a long-term desire to study medicine,

‘Yes when I was still in high school, I had a dream of becoming a doctor.’ (Doctor Q, female, 46, 10 July 2017)

‘It was my childhood dream to become a doctor.’ (Doctor P, female, 41, 07 July 2017)

#### Altruism or wanting to help people and the community:

Others wanted to help people and/or the community,

‘Well that- it’s a long story but you know in short I always loved working with people, I always loved trying to help which is what I felt in a way if I was a doctor I could possibly do that.’ (Doctor E, male, 36, 14 June 2017)

‘To help the community, especially the poor community, in the deep rural areas like this one, like Nkandla to help service this.’ (Doctor N, male, 41, 05 July 2017)

‘I had a major accident and I was involved in a car accident and I had multiple femur fractures with an ankle fracture … um I was taken, and my family taken [me] to a hospital in the Limpopo … I think it was Groblersdal Hospital … and unfortunately that hospital did not have an X-ray machine, um I remember that night. We waited quite a while, you know, to tend to all of us because there was shortage of doctors and there was just one doctor, if I remember correctly - um … in casualty that tended to us …’ (Doctor B, male, 30, 28 May 2017)

#### Role models

For some participants, the desire to pursue medicine came after having been exposed to a role model who was a doctor.

‘… one being my uncle, he was a doctor. I fell in love with the idea of working for myself …’ (Doctor G, male, 41, 22 June 2017)

‘No it was because you know when you grow up in the rural area, there was only 1 doctor- his name was Dr. X and he was the guy that he was the only person that had by that time the only guy that had a degree in the place but he was not from that village- he would naturally come to work at the nearest hospital, and he then opened up his own business and we looked up to Dr. X, actually he was one of the guys that made me think that actually it’s nice to be a doctor …’ (Doctor H, male, 35, 22 June 2017)

### Professional career decisions

All the doctors had completed the compulsory internship year. Nineteen had done so between 2004 and 2010 and the most recent recruit did so in 2015. While most of the doctors (*n* = 15) had completed the internship period in KZN, two did their internship in Gauteng and three had done so in Mpumalanga.

Most participants (*n* = 19) completed community service over the 6-year period 2005–2011 and one had embarked on community service in 2017. Most of the doctors completed their community service in rural areas of SA. Seventeen completed their community service in KZN, one in Northwest province and two in Mpumalanga.

Rural obligation: For all the doctors in this study, this meant an obligation to work in an underserved area which equalled the duration of their medical training, because of the contract they had signed at the onset of their Cuban medical training. Nineteen participants had already honoured the obligation. One doctor was in the process of completing this obligation. When asked about their intentions to work elsewhere, their comments indicated that all the participants had valued the experience and had planned to honour their commitment regardless of the contractual agreement. As indicated below, some regarded service to their communities as an obligation that they would have fulfilled irrespective of any contract:

‘I think it was something I would have done anyway, because I want to …’ (Doctor L, male, 40, 05 July 2017)

‘I want to do work in areas that are disadvantaged- work at places where others don’t want to work …’ (Doctor Q, female, 46, 10 July 2017)

‘Yeah, I had to honour a contract and again it was crucial at the end of the day that I give back to the community, I have a community that is [in] need of medical care.’ (Doctor C, male, 34, 05 June 2017)

As a condition of the scholarship, recipients were obliged to repay in kind by providing clinical services at PHC facilities in their country of origin and for a period that equates to the time for which the scholarship had been received.^30^ In terms of the obligation to work in rural or underserved areas, 11 doctors were still in practice in a rural location at the time of data collection. The career choices of the participants following their graduation from the programme are outlined in Table 2.

**TABLE 2:** Details of career choices of participants (*n* = 20).

Unique participant identifier	Role	Practice type	DoH level of service	Private	NGO/other	Province	Location
1	GP/MO	PHC	Sessions: district hospital	Part time	-	KZN	Semi-urban
2	MO	PHC	District hospital	-	-	LP	Rural
3	GP	PHC	Sessions district hospital	Part time	-	KZN	Semi-urban
4	Freelancing GP (locum basis)	PHC/male medical circumcision	-	-	PHC	KZN	Rural
5	MO/GP	PHC	District hospital	Part time	PHC	KZN	Rural
6	Clinical manager	PHC	District hospital	-	-	NW	Rural
7	CEOADMIN	PHC	District hospital	-	-	NW	Rural
8	Registrar	Orthopaedics	Tertiary hospital	-	-	GP	Urban
9	Administrative head office	Policy	Governmental national	-	-	GP	Urban
10	MO	PHC-PSYCH	District hospital	-	-	KZN	Semi-urban
11	MO	PHC	District hospital	-	-	KZN	Rural
12	GP	PHC	-	Solo private	-	KZN	Rural
13	GP	PHC	-	Solo private	-	KZN	Urban
14	MO	PHC	District hospital	-	-	KZN	Rural
15	Consultant specialist	Orthopaedics	Private hospital	Solo private	-	KZN	Semi-urban
16	Clinic manager Medical manager	PHC manager	District hospital	-	-	KZN	Rural
17	MO	PHC	District hospital	-	-	KZN	Rural
18	MO/GP	PHC	District hospital	Part time	-	KZN	Rural
19	Consultant	Obstetrics and gynaecology	Tertiary hospital	-	-	KZN	Urban
20	Consultant	Psychiatry	Tertiary hospital	-	-	GP	Urban

MO, medical officer; GP, general practitioner; NGO, non-governmental organisation; DoH, South African Department of Health; KZN, KwaZulu-Natal; MP, Mpumulanga; NW, North West; LP, Limpopo; PHC, primary healthcare.

In general, the participating doctors were practicing in KZN (*n* = 14), Gauteng (*n* = 3), the Northwest province (*n* = 2) and in Limpopo (*n* = 1). While 13 doctors were working in PHC, there were three doctors who had specialised (psychiatry, obstetrics and gynaecology and orthopaedic surgery). Two of the specialists were working for the DoH and one of the specialists was in private practice. Two doctors were employed as medical managers in the DoH, one doctor was employed by the national government and one was still enrolled in specialisation training at the time of data collection. The PHC practitioners were either employed by the DoH (*n* = 9), in solo private practice (*n* = 2) or employed at a non-governmental organisation (*n* = 2).

Nineteen doctors indicated an intention to work and live within South Africa in the long-term. Two specialists, both of whom were attached to the public sector, intended to work in private practice. The specialist who was in private practice at the time indicated an intention to remain in private practice, while those in general practice also wanted to build their private practices. The medical officers (*n* = 9) who were in public sector service wished to continue working in the public sector. Three of these doctors mentioned plans to specialise either in surgery, family medicine, pathology, psychiatry or anaesthesia.

Two doctors wanted to pursue specialisation in public health, while one mentioned a desire to work abroad and the other wanted to do so in the SA health policy context. A registrar who was specialising at the time of data collection indicated an intention to pursue work in his home community. Most of the doctors (*n* = 11) had no desire to practice abroad or to emigrate from South Africa. Nine participants wanted to gain international experiences including working on a cruise ship, another in Switzerland for 5 years, pursuing overseas study, while one doctor wanted to work in a Spanish speaking country. Eight of the nine doctors indicated an intention to return to SA in the long-term. Only one doctor wanted to emigrate from SA permanently.

## Discussion

The findings highlight that all the graduates (*N* = 20) who participated in this study and who were recipients of the SACMC programme have returned to local SA settings. All of the participants preferred to enrol on the SACMC programme as it was the only scheme at the time to offer them a full scholarship to pursue medical training. Nineteen doctors had fulfilled their obligation to work in rural areas. Thirteen doctors are engaged in PHC practice, either as private practice generalists or as public service medical officers. Three doctors had completed and one was still training towards a specialisation. One of the doctors was employed at a governmental level, and two doctors were employed as medical managers. At the time of the study, 11 doctors were practicing in rural locations. Nineteen of those interviewed had indicated a long-term intention to continue working and liveing within South Africa.

As the SA government is trying to find suitable and sustainable long-term options to provide development and training to SA’s large uneducated youth population, many of whom are from rural and non-fee paying schools,^31^ the current case shows how the SACMC collaboration programme served as a viable option to educate and professionalise a particular cohort of 20 scholarship recipients. The programme offered these participants the only option to pursue a career that they perceived as rewarding and that fulfils South Africa’s need to provide more doctors for PHC in rural areas. Participants in our study chose medicine as a career citing reasons that include the opportunity for free education, the presence of an inspirational role model, the pursuit of a childhood dream and/or the desire to help people. These reasons are similar to those cited by junior doctors in a study from Sierra Leone, a low-income country which similarly struggles to recruit and retain its healthcare workforce.^32^

Most of the participants have returned to a rural area to fulfil their scholarship obligation and 11 of the participants have continued practicing in a rural area at the time of the study. This is of interest as the rural areas of SA are areas where there is the greatest need for doctors to serve the large number of patients with a high burden of disease.^2^ While 70% of the doctors in this study originated from KZN, there were also representatives from three other provinces. The findings of our study resonate with that of Ross and colleagues’ review of the Umthombo Youth Development Foundation scholarship scheme.^33^ That study reported on the period from 1999 to 2013 where 430 students of rural-origin received scholarships to study across 15 health science disciplines. Their report indicates that all graduates had spent time working in rural areas and that 72% of students who had completed their rural work-back obligation had continued their careers in rural sites.^33^

All of the doctors in this study originated from a rural and disadvantaged background and perceived that they faced greater limitations and fewer career options because of their socio-economic backgrounds. The sentiments expressed by participants in our study about limited career options to students from a rural-origin still reflect the findings of a study that was conducted 10 years ago by Tumbo et al.^34^ That study of more than 7000 undergraduates from nine health science faculties in South Africa found the proportion of rural-origin students enrolled at health science institutions to be lower than the national rural population ratio.^34^ It is very likely that these ratios as reported in health science faculties have not improved and further confirmatory research in this areas is needed.

South African institutions of higher learning are furthermore challenged by the ‘FeesMustFall’ student unrest which was largely sparked by the increases in student fees and limited access of students to tertiary education. This problem is largely intensified for rural students who generally have limited access to information about careers and ways to finance their studies.^32^ While rural education is generally under-resourced and inadequate, a study with students from rural KZN has shown that those enrolled in tertiary studies can be successful when provided with sufficient financial and moral support.^35,36^ The study also recommended for the recruitment and selection of students to such programmes to consider students’ personal attributes including determination, problem-solving ability, self-belief and commitment to hard work, as these attributes had been influential indicators of success and positive career outcomes.^36^

Nineteen doctors had fulfilled their obligation to work in rural areas. Their comments indicated that the decision to work in underserved areas was not only because of their contractual obligations but also for altruistic reasons. While it is possible that other recipients of the SACMC scholarship might have neglected their rural obligations, moved to urban areas, emigrated from South Africa or selected to practice in private settings, our findings show that this is not the case as there are at least 11 of these Cuban-trained doctors who are still providing PHC services to communities in rural areas. This finding also supports that of other studies which showed that there are still healthcare practitioners practicing in rural areas of SA who do so for altruistic reasons.^3^ These findings are important as the retention of doctors to rural areas is required to alleviate the critical shortage in the medical workforce.^2^

Most of the doctors in this study also preferred to practice in the PHC sector and 11 doctors were still practicing in a rural location. The career choices of the 20 doctors suggest that they were free to choose their preferred practice location (rural vs. urban). They felt free to change the nature of their medical practice (specialist, general practice or administrative positions) and the type of practice (public sector vs. private sector). The 20 participants furthermore did not perceive any limitations to their careers because of obligations of the SACMC scholarship. The graduates are fulfilling their full potential and felt free to pursue all career aspirations irrespective of having trained in Cuba. They were essentially pursuing the same options as their locally trained counterparts but with different end points.

Our study involved participants who graduated from the SACMC, and therefore differs from studies that had been conducted on subsets of undergraduate students who had returned to SA to complete their training at local institutions^21,37,38^, and Báez’s description of the experiences of Cuban citizens as physicians in the Gauteng province.^39^ Our findings are also markedly different from that of Bezuidenthout et al. who reported on a sample of 27 local SA-trained graduates who had predominantly been working in private practice in SA settings before emigrating.^40^ In that study, 86.2% of the sample identified finances as their main reason for emigrating from SA and 75% said that they would recommend emigration to newly SA-trained doctors.^40^ From the above, it is clear that most studies had been conducted with undergraduate students of the SACMC programme and the current study therefore presents new information on the career choices and career intentions of a sample of practicing doctors who were beneficiaries of the programme.

One of the aims of the SACMC was also for graduates as qualified doctors to use their knowledge of the Cuban PHC model to transform and contribute to the re-engineering of the SA public health system.^15,17^ The degree to which these graduates actually apply the PHC principles in clinical practice has yet to be verified and was outside the scope of the current study. Further research will help to determine whether the Cuban-trained doctors are practicing differently to their SA-trained counterparts and whether local healthcare workers are actually learning from them. It is also anticipated that an integrated policy and firm commitment to the NHI may influence the role that SACMC graduates can play in the PHC re-engineering process.

### Limitations

This qualitative study of 20 participants of the SACMC programme was intended to improve our knowledge of their reasons for selecting to study on the programme and their career choices and intentions since graduation. The exploratory case study design was deemed as an appropriate approach to answer the research objectives. The findings are therefore peculiar to only these participants and no attempt has been made to generalise the findings to other recruits of the SACMC.

While the purposive sampling strategy was useful to gather perceptions of all beneficiaries of the collaboration during the specified period, the database proved outdated. The resultant use of the snowball strategy to increase the number of participants could furthermore have skewed the findings as most of the participants originated from KZN. As the findings reflect the perceptions of a small sample, it is necessary that future studies include greater representation of participants from all the participating provinces and that a national quantitative study be designed to determine the long-term outcomes of the SACMC in the SA context. Research into the social and professional challenges of returning doctors upon reintegrating into the SA healthcare settings would also be beneficial. It would also be useful to learn whether their stronger PHC orientation has had an impact on the health systems in which they work.

## Conclusions

Twenty disadvantaged students from rural areas of SA received an opportunity to study medicine in Cuba. All the doctors are contributing to SA’s medical workforce. Most of the doctors reported having a fulfilling medical career and expressed a long-term desire to contribute to the SA healthcare system. The findings of this case study are useful as they describe the career choices and intentions of a subset of SA beneficiaries of the SACMC programme. It also identified the need for further academic discourse in this area.
